# Covering loyalty policy in quiet firing workplace: the association between quiet quitting, intention to leave, and nurses’ loyalty

**DOI:** 10.1186/s12912-025-03301-8

**Published:** 2025-06-20

**Authors:** Ahmed Abdellah Othman, Hossam Mohamed Mahran, Hind Ismail Ali

**Affiliations:** 1 Department of Nursing Administration, Faculty of Nursing, Sohag University, Sohag City, Egypt; 2 Department of Nursing, College of Applied Medical Sciences in Wadi Addawasir, Prince Sattam Bin Abdul Aziz University, Wadi Alddawasir, Saudi Arabia

**Keywords:** Nurses, Intention to leave, Loyalty, Quiet firing, Quiet quitting

## Abstract

**Background:**

Nurses’ intention to leave harms healthcare organizations and the nursing profession. Organizational productivity level that cannot be achieved without their attention to improving nurses’ loyalty with quiet firing management and nurses’ quiet quitting.

**Purpose:**

This study aims to examine the relationships between nurses’ loyalty, intention to leave, quiet quitting, and quiet firing. Also, investigate the role of quiet quitting in the relation between nurses’ quiet firing, loyalty, and intention to leave.

**Methods:**

The study employed a cross-sectional design. Data were collected from nurses in Sohag University Hospital, Egypt. It was conducted with 482 nurses who had worked at their employing facility. Researcher used three scales; intention to leave scale, loyalty scale and quiet quitting and quiet firing scale.

**Results:**

Shows that there was a high statistically significant (*P* < 0.001) positive correlation between quiet quitting intention and perceived quiet firing (*r* = 0.460**), quiet quitting intentions and intention to leave scale (*r* = 0.464^**^), perceived quiet firing and intention to leave scale (*r* = 0.450^**^), while there was a statistically significant negative correlation between nurses’ loyalty and quiet quitting and quiet firing scale at (*r* = -0.300^**^) and nurses’ loyalty and intention to leave scale at (*r* =-0.186^**^).

**Conclusion:**

The research findings concluded that there was a highly statistically significant relation between quiet quitting intentions, perceived quiet firing, nurse loyalty, and intention to leave. Also, there was a statistically significant indirect effect of perceived quiet firing on nurses’ intention to leave and perceived quiet firing on nurses’ loyalty when the perceived quiet firing acted as a mediator variable.

**Implications for nursing and health policy:**

Policy implications to increase nurses’ loyalty by increasing nursing participation in hospital committees, promotion opportunities, implementation of professional practice models, and use of mentorship programs, to competitive compensation and career development opportunities.

## Introduction

Healthcare workers, particularly nurses, face significant rates of burnout, discontent, anxiety, and depression as a result of the demanding and stressful nature of providing [1]. To meet the requirements of their patients, nurses suffer from enthusiasm, devotion, empathy, and an incredible amount of concern [2]. The managers need to guarantee a productive workplace for nurses over time while conserving the quality of nurses’ well-being and, sufficient staffing of nursing [3].

Nurses are supposed to be “loyal” to their values and goals as individuals and as clinicians, as well as to their patients, employers, and the nursing profession. To describe how nurses become loyal to their organization using the attribution theory, where a person’s actions are influenced by internal (nurses’ ability) and external strengths(work environment) [4]. Internal attribution, which is impacted by nurses’ motivation, dedication, and contentment, includes nurse loyalty [5]. Employee loyalty can be seen as an attitudinal inclination toward identification and trust in the organization. Loyalty is a characteristic visualized in terms of person–workplace interaction [6].

The nursing work environment, encompassing accessible resources and established processes, might affect nurses’ loyalty. Loyalty is the degree to which employees feel committed to the organization and intend to stay long-term. Organizational loyalty among nurses has been traditionally associated with factors such as positive relationships with supervisors, adequate compensation, career growth opportunities, and supportive work environments [7]. However, when these factors are absent or diminished, due to phenomena like quiet quitting or quiet firing, nurses’ loyalty significantly declines. Research indicates that employees who experience negative organizational practices (like quiet firing) are less likely to feel emotionally attached or loyal to the organization [8].

The organizational climate promotes open communication to encourage nurses to voice their ideas. Nurses who are unable to express feelings and ideas and cannot be listened to fairly will tend to leave. Employees choose not to invest more time, energy, or emotion in their work than is strictly required as a result of this silence [9]. Employees decide to quit due to job discontent, lack of commitment to the company, job-related stress, and other variables that contribute to job-related stress [10]. Employee withdrawal by exhibiting low work engagement and dissatisfaction against workplace issues such as stress, anxiety, workload, and lack of support is called quiet quitting [13]. It occurs when an employee stays with an organization for less than a year before exiting. A quiet quitter will not participate in or start activities that involve the free exchange of knowledge, such as attending integrative events or organizational meetings [11].

Factors contributing to nurses’ quiet quitting (QQ) include workplace environment challenges and inadequate management practices, such as bullying and horizontal aggression, along with a failure to acknowledge and adequately reward the contributions of the most productive nurses. Nurses who engage in quiet quitting are more prone to express turnover intentions [12]. Additionally, some scholars claim that hustle culture is the reason behind quiet quitting [13–15]. According to some researchers, QQ represents the behavioral response of employees to a culture of constant change and high demands within organizations. In response to this, organizations may exhibit the phenomenon known as Quiet Firing (QF) [13]. Quiet firing is a technique that can range from the manager’s (intentional) failure to provide an employee with enough coaching, support, and career development to the manager creating genuinely toxic and miserable working conditions for an employee, which is a type of gaslighting [16, 17].

While quiet quitting focuses on employee behavior, quiet firing reflects organizational actions. Quiet firing occurs when employers use subtle, non-direct methods, such as increased workload, lack of recognition, or unfavorable job conditions, to push employees to leave voluntarily. This tactic is often employed to avoid legal ramifications and the cost of formal layoffs [18]. In nursing, quiet firing might manifest as a lack of professional development opportunities, exclusion from decision-making, or removal from preferred shifts or roles, leading to a decline in nurses’ job satisfaction and loyalty [19, 20].

Nurses subjected to quiet firing are more likely to experience lower levels of job satisfaction and organizational loyalty, leading them to consider leaving the organization or the profession entirely [21]. A recent study revealed that 67.4% of nurses were quiet quitters, while the prevalence of quiet quitting for physicians and other HCWs was 53.8% and 40.3%, respectively. Lower satisfaction levels among nurses may result in increased turnover intentions, indicating poor organizational loyalty [22].

The dynamic between quiet quitting, quiet firing, and nurse retention is complex. Quiet quitting may lead to lower performance and decreased job satisfaction, but it does not necessarily result in immediate turnover. However, if quiet firing is perceived by nurses as an intentional strategy by the employer to push them out, it can amplify their intention to leave, eroding loyalty over time [23]. A study on employee retention in the nursing field found that organizational loyalty was directly linked to the intention to stay [24]. Conversely, employees who experienced conditions such as underappreciation or lack of career advancement (hallmarks of quiet firing) exhibited stronger intentions to leave. This behavior was also influenced by job stress and burnout, which are common triggers for both quiet quitting and the perception of being subjected to quiet firing [25].

Leadership plays a critical role in preventing quiet quitting and quiet firing and fostering nurse loyalty. Transformational leadership, which emphasizes support, communication, and professional development, has been shown to improve organizational commitment and reduce turnover intention in nursing [26]. Conversely, poor leadership that fails to address employee concerns or provides insufficient support can exacerbate the quiet quitting and firing cycle, leading to a vicious cycle of disengagement and eventual turnover [27].

In 2021, Egypt counted 192,086 workers in the nursing staff of the governmental healthcare sector. This was a decline from the previous year. However, the nursing staff amounted to 161,717 workers in 2011 and saw a gradual increase over the years [28]. The decline in the number of nurses is a disturbing issue for nurse managers to achieve the National Health Vision 2030. In this context, the study aimed to examine the relationships between nurses’ loyalty, intention to leave, quiet quitting, and quiet firing. Also, investigate the role of quiet quitting on the relation between nurses’ quiet firing, loyalty, and intention to leave.

### Theoretical framework

Understanding the relationship between quiet quitting, intention to leave, and nurses’ loyalty would likely involve an intersection of social exchange theory [29] Organizational commitment theory [30], and the Job Demands-Resources (JD-R) model [31] might provide a comprehensive understanding of how organizational conditions, employee engagement, and satisfaction influence nurses’ work behavior, loyalty, and retention. The current study builds the hypotheses on the JD-R model, positing that job demands (stressors, workload, and emotional strain) can lead to burnout and disengagement, while job resources (support, autonomy, recognition) can buffer stress and foster engagement. Quiet quitting could be linked to high job demands without sufficient resources, leading to a disengaged workforce. On the other hand, when nurses feel supported and valued, they are more likely to exhibit higher levels of loyalty. Quiet quitting might occur when nurses are overwhelmed by job demands with limited support. Nurses’ intention to leave could arise when resources do not meet demands over time. Loyalty, in turn, might be fostered when there is a balance between job demands and resources, leading to higher engagement and retention.

## Methods

### Purpose

The purpose of this study is to examine the relationships between nurses’ loyalty, intention to leave, quiet quitting, and quiet firing. Also, investigate the role of quiet quitting in the relation between nurses’ quiet firing, loyalty, and intention to leave.

### Research hypotheses figure (1)

#### H1

Nurses’ loyalty is negatively correlated with quiet firing.

#### H2

Nurses’ loyalty is negatively correlated with quiet quitting.

#### H3

Nurses’ loyalty is negatively correlated with intention to leave.

#### H4

Quiet quitting has a mediating role in the relation between nurses’ quiet firing, loyalty, and intention to leave.

**Fig. 1:**
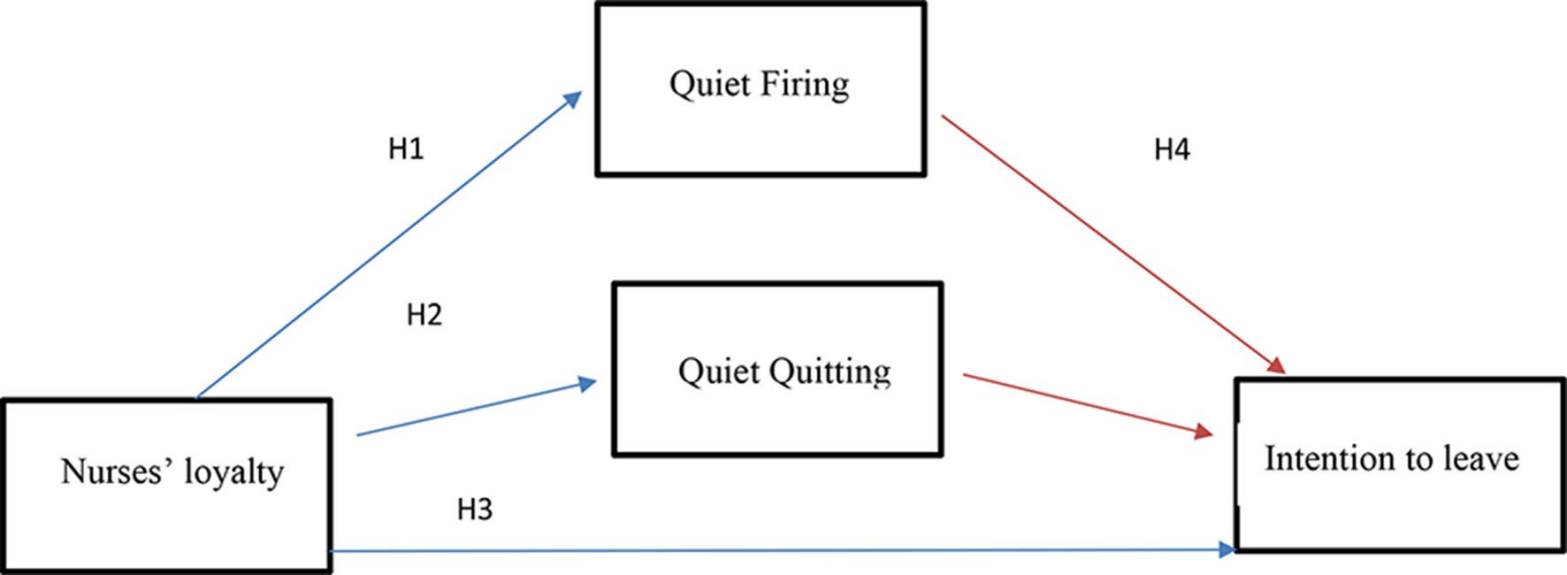
The research hypotheses

### Study design, setting, and participants

This researcher utilized a descriptive cross-sectional design. The study was located at the Sohag University Hospital in Sohag City, Egypt. The target population for this study was 482 nurses who worked at the previously mentioned setting. The Strengthening the Reporting of Observational Studies in Epidemiology (STROBE) guidelines were followed. The 22-item STROBE checklist provides key reporting recommendations for each section of the manuscript, including the title, abstract, introduction, methods, results, and discussion.

In the power analysis performed with G*Power software, that utilized to determine the study’s sample size with 482 nurses were required to attain 0.99 Power with an Effect size d = 0.2, Alpha 0.05, and a two-tailed test for Means: Wilcoxon signed-rank test, The total number of nurses needed according to G*Power software was 482. The inclusion criteria for the participating nurses include nurses who have current clinical employment, at least 6 months of work experience in nursing wards, and who give their voluntary consent.

### Measurement

#### Personal data form

It included data such as nurses’ age, gender, educational level, place of residence, marital status, years of experience, and perception of monthly income.

#### Loyalty Scale

It was developed by Dutta & Dhir (2021) [6] to evaluate the loyalty status of the employees toward their organization. It was translated into Arabic by the researchers. There were thirteen parts in all, divided into three dimensions: ownership (6 items), willingness to stay (3 items), and trust (4 items). The participants were provided with a 5-point Likert scale to indicate whether they agreed or disagreed with the statements on the scale. The responses ranged from “Strongly agree = 5” to “Agree = 4” to “Uncertain = 3” to “Disagree = 2” to “Strongly disagree = 1.” Each dimension’s results were added up and then expressed as a percentage score. The reliability coefficient value was 0.79 [6].

#### Quiet quitting and quiet firing scale

It was adopted from Karadas & Çevik (2024) [32] to assess the employees’ quiet quitting intentions and perceived quiet firing. It contained 14 items divided into two dimensions: quiet quitting intentions (7 items), such as “I often avoid working more hours if there is no additional pay,” and Perceived quiet firing (7 items), such as “My manager/supervisor gives me limited time off from work.” The participants` responses were on a five-point Likert-type scale (1 = never/completely disagree; 5 = very often/completely agree). The Cronbach’s ⍺ internal consistency coefficient was 0.89 [32].

#### Intention to leave scale

It was employed by Meyer et al. (1993) [33] to assess intention to leave the organization with 3 items: First, how frequently the employee thinks about leaving his or her current employer; Second, how likely it is that the employee will search for a job in another organization; and Third, how likely it is that an individual will leave the organization within the next year. The responses were taken on a 7-point Likert scale as follows: [1] strongly disagree [2], disagree [3], slightly disagree [4], neither agree nor disagree [5], slightly agree [6], agree, and [7] strongly agree. The 3-item intention to quit scale had a high coefficient of validity = 0.89 [33], and reliability was = 0.71 [34].

### Data collection procedures

Data for the study were collected in 2024 between August and December. To make sure the used tools were valid and reliable, a pilot research study was carried out with 48 nurses to evaluate the instruments’ reliability, relevance, and intelligibility. Internal consistency was confirmed by using statistical techniques to assess reliability, such as Cronbach’s alpha. A pilot study participant was included in the main investigation, as these findings demonstrated that no changes were necessary and validated that the instruments were suitable for the primary research.

The process of data collection began after obtaining the necessary authorizations and protecting excel documents with data on each nurse from the hospital’s human resources department. The participants were selected using a random number generator, and the process was repeated until each unit had the required number of nurses. Prior to data collection, researchers provided each nurse with a detailed explanation of the study objectives, emphasizing that participation was entirely voluntary.

Informed consent was obtained from all participants as a prerequisite for their involvement. Researchers reassured participants that their answers would be kept private in order to maintain confidentiality and build confidence. According to the workload of the units from Saturday through Thursday, questionnaires were handed out in various shifts in the morning, evening, and night. On average, participants spent 15 to 20 min completing each questionnaire.

According to Table (1): Cronbach’s alpha coefficients for the Intention to leave were 0.76, Quiet quitting and quiet firing was 0.76 and Loyalty Scale was 0.83calculated to assess internal validity, with values ranging from 0.808 to 0.943. Composite reliability (C.R.) values ranged from 0.76 to 0.83, and the average variance extracted (AVE) values ranged from 0.52 to 0.68.

**Table 1:** Measurement model fit indices for tools

Fit Indices	Indicators		
	Intention to leave	Quiet quitting and quiet firing	Loyalty Scale
Chi-square	261.507 (*p* < 0.001)	171.327 (*p* < 0.00)	293.692 (*p* < 0.000)
Chi-square/df (degree of freedom)	0.959	0.886	0.825
Goodness of Fit Index (GFI)	0.928	0.9164	0.876
Adjusted Goodness of Fit Index(AGFI)	0.968	0.905	0.752
Relative Fit Index (RFI)	0.963	0.976	0.955
Normed Fit Index (NFI)	0.971	0.871	0.973
Comparative Fit Index (CFI)	0.068	0.041	0.074
Root Mean Square Error of Approximation (RMSEA)	0.049	0.0536	0.0343
Average Variance Extracted(AVE)	0.56	0.68	0.52
Reliability	0.76	0.91	0.83
Composite Reliability (CR )	0.81	0.76	0.83

### Data analysis

SPSS 26.0 (IBM Inc., Chicago, IL, USA) software program was used for data analysis in order to assess the 482 nurses’ survey answers. Descriptive statistics were used to characterize the overall characteristics of the participants and the scores on different measures. Categorical variables were shown in number and percentage and numerical variables were shown in mean ± standard deviations. To evaluate correlations between research variables, Pearson’s correlation analysis is used. The mediating function of quiet quitting intentions between loyalty, intention to leave, and perceived quiet firing was tested using JASP 0.14.1.0. Statistically significant was interpreted at the level of *P* ≤ 0.05.

### Ethical considerations

The study was ethically approved by the Research Ethics Committee of the Faculty of Sohag University (Serial No: 182) in August 2024. Because the study conformed with all relevant laws, local regulations, and the ethical norms of the Declaration of Helsinki, the participants’ rights and safety were safeguarded. The researchers obtained informal consent from all participants. The participants were given a thorough explanation of the study’s objectives and were reassured that their involvement would be entirely voluntary and private. They were given guarantees that all collected data would be kept private and that only the authorized study team would be able to access it. Each participant provided written informed consent before any data collection.

## Results

**Table 2:** Personal characteristics among participants (*n* = 482)

Personal data	Categories	No	%
Age	< 25	81	16.8
	25 < 35	343	71.2
	35 ≥ 40	58	12.0
Gender	Male	92	19.1
	Female	390	80.9
Marital status	married	386	80.1
	Unmarried	96	19.9
Education level	Diploma / Nursing Technical Institute	347	72.0
	Bachelor’s degree in nursing	119	24.7
	Postgraduate education	16	3.3
Years of experience	1 < 5 yrs.	171	35.5
	5 ≤ 10 yrs.	289	60.0
	< 10 yrs.	22	4.6
Place of residence	Urban	185	38.4
	Rural	297	61.6
Perception of monthly income	Enough	161	33.4
	Not enough	321	66.6

### Description of participant nurses

The study included 482 participants nurses with 71.2% of the participants were aged 25–35 years old, 80.9% of them were females, 80.1% of them were married, 72.0% of them had a diploma from a nursing technical institute, 60% of them had 5–10 years of experience in nursing, 61.6% of them were from rural areas, and 66.6% of them didn’t have enough monthly income. Table (2).

**Table 3:** Descriptive analysis of study variables (*n* = 482)

Variables	Mean ± SD	Average Mean ± SD
Quiet Quitting Intentions	21.330 ± 6.938	3.047 ± 0.991
Perceived quiet firing	21.201 ± 8.470	3.029 ± 1.210
Quiet Quitting and Quiet Firing scale	42.512 ± 13.401	3.037 ± 0.957
Loyalty	23.799 ± 6.238	1.831 ± 0.480
Intention to leave scale	15.687 ± 2.644	5.229 ± 0.881

SD = Standard deviation

### Study variables description

Table (3): Reveals that the average mean score of the participants on the quiet quitting and quiet firing scale is 3.037 ± 0.957, with the subscales being 3.047 ± 0.991 for quiet quitting intentions and 3.0287 ± 1.210 for perceived quiet firing. Furthermore, the average mean score of the participant nurses’ loyalty scale was 1.831 ± 0.480, and the average mean score of the participants’ intention to leave scale was 5.229 ± 0.881.

**Table 4:** Correlation between study variables among participants (*n* = 482)

Variables	1	2	3	4
Quiet Quitting Intentions	1			
Perceived quiet firing	0.460^**^ (< 0.001)	1		
Quiet Quitting and Quiet Firing scale	0.491^**^ (< 0.001)	0.540^*^ (< 0.001)	1	
Loyalty	− 0.300^**^ (< 0.001)	− 0.289^**^ (< 0.001)	− 0.198-(< 0.001)	1
Intention to leave scale	0.464^**^ (< 0.001)	0.450^**^ (< 0.001)	0.318^**^ (< 0.001)	− 0.186-^**^(< 0.001)

r = Pearson correlation, ** correlation is highly significant at the 0.01 level (2-tailed)

### Correlation between study variables

**Fig. 2:**
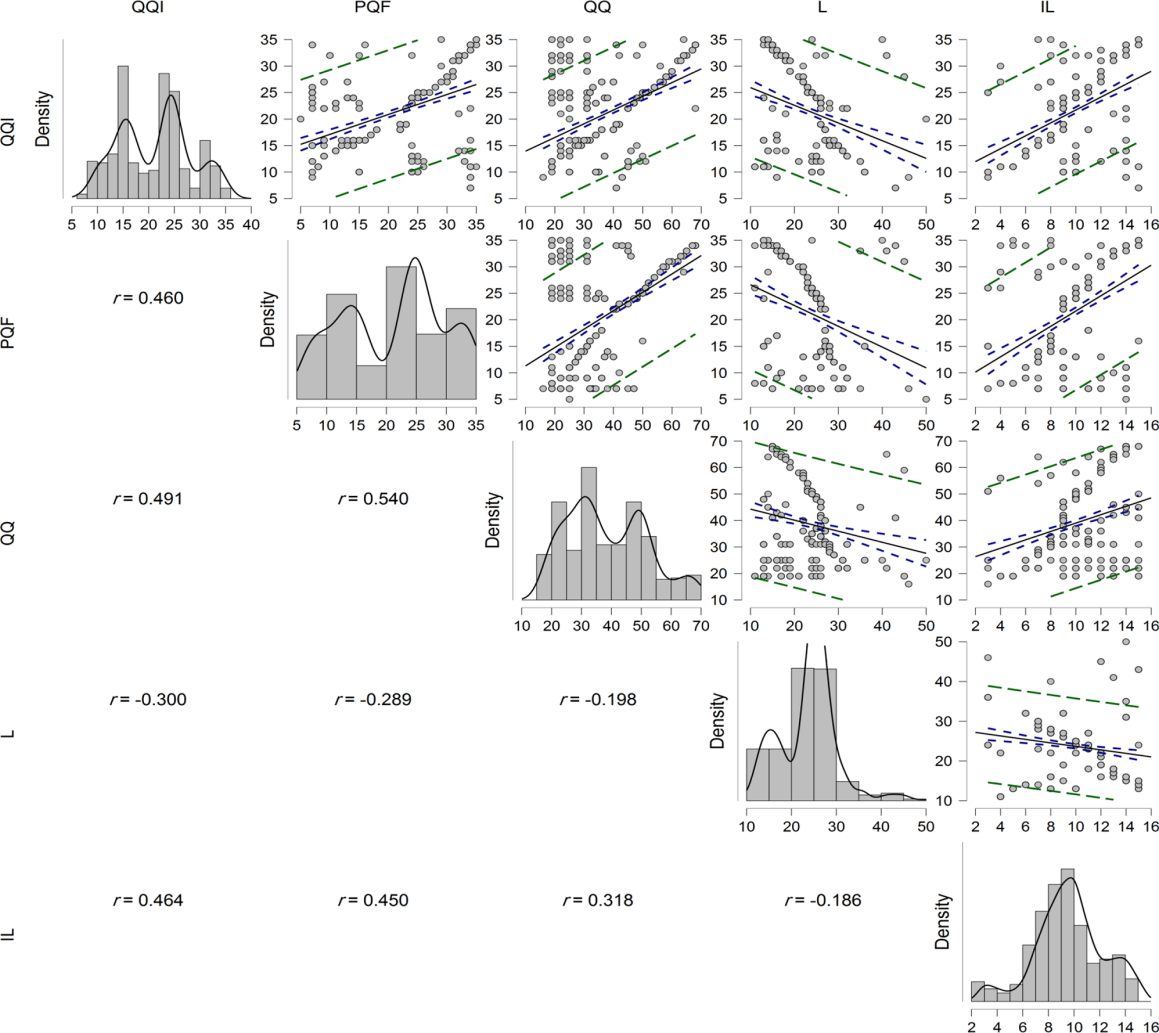
Density and correlation between study variables. PQF, Perceived quiet firing; QQI, Quiet Quitting Intentions; I, Intention to Leave; ll, Loyalty

There was a medium statistically significant positive correlation between quiet quitting intentions and perceived quiet firing (*r* = 0.460^**^, *P* < 0.001), quiet quitting intentions and quiet quitting and quiet firing scale (*r* = 0.491^**^, *P* < 0.001), perceived quiet firing and quiet quitting and quiet firing scale (*r* = 0.540^**^, *P* < 0.001), quiet quitting and quiet firing scale and intention to leave scale (*r* = 0.450^**^, *P* < 0.001), quiet quitting intentions and intention to leave scale (*r* = 0.464^**^, *P* < 0.001), perceived quiet firing and intention to leave scale (*r* = 0.450^**^, *P* < 0.001), while there was a low statistically significant negative correlation between nurses’ loyalty and quiet quitting and quiet firing scale at (*r* =-0.198^**^, *P* < 0.001) and nurses’ loyalty and intention to leave scale at (*r* =-0.186^**^, *P* < 0.001). Table (4) & Figure (2).

**Table 5:** Path analysis for the effect of quiet quitting intentions between perceived quiet firing, loyalty, and intention to leave (*n* = 482)

Direct effect	(B)	CI 95%	t	*p*
Perceived quiet firing → Intention to leave	0.094	(0.067 --0.120)	6.919	< 0.001^**^
Perceived quiet firing → Loyalty	-0.141	(0.210 − 0.071)	-3.970	< 0.001^**^
Perceived quiet firing→ Quiet Quitting Intentions	0.377	(0.311-0.442)	11.340	< 0.001^**^
Quiet Quitting Intentions→ Intention to leave	0.177	(0.147-0.207)	11.476	< 0.001^**^
Quiet Quitting Intentions → Loyalty	− 0.270	(-0.347–0.193-)	-6.898-	< 0.001^**^
**Indirect effect**				
Perceived quiet firing → Quiet Quitting Intentions → Intention to leave	0.047	(0.032–0.061)	6.276	< 0.001^**^
Perceived quiet firing → Quiet Quitting Intentions → Loyalty	-0.072	(-0.106 -0.038)	-4.114	< 0.001^**^
**Total effect**				
Perceived quiet firing → Intention to leave	0.140	(0.116–0.165)	11.056	< 0.001^**^
Perceived quiet firing → Loyalty	-0.213	(-0.276 -0.150)	-6.622	< 0.001^**^

**Fig. 3:**
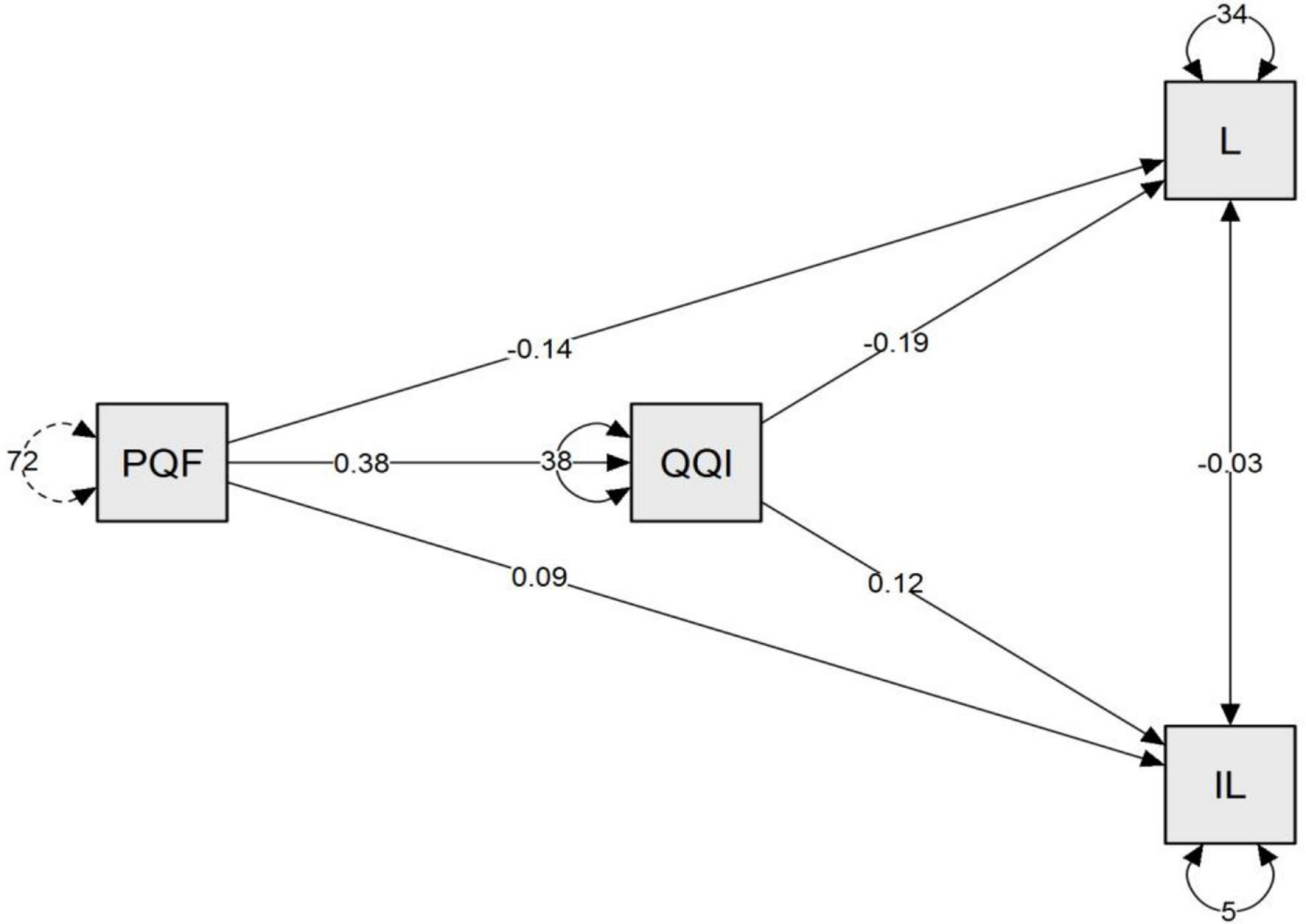
Mediating model effect of Quiet Quitting Intentions between Perceived quiet firing, Loyalty, and Intention to leave (*n* = 482). PQF, perceived quiet firing; QQI, Quiet Quitting Intentions; IL, Intention to leave; L, Loyalty

### Mediation analysis

Table (5) & Figure (3): Illustrate that, there was a high statistically significant direct effect of perceived quiet firing on intention to leave at (B = 0.094, t = 6.919, < 0.001), on nurse loyalty at (B=-0.141, t=-3.970, < 0.001), perceived quiet firing on participant nurses’ quiet quitting intentions at (B = 0.377, t = 11.340 < 0.001), quiet quitting intentions on intention to leave at (B = 0.177, t = 11.476 < 0.001), quiet quitting intentions on loyalty at (B=-0.270, t=-6.898- < 0.001). Furthermore, there was a highly statistically significant indirect effect of perceived quiet firing on nurse intention to leave at (B = 0.140, t = 11.056, < 0.001), perceived quiet firing on nurse loyalty at (B=-0.213, t=-6.622, < 0.001) when the perceived quiet firing acts as a mediator variable.

## Discussion

The average mean score of the participants’ quiet quitting and quiet firing scale was neutral, with the subscales for quiet quitting intentions and perceived quiet firing. Accordingly, with these results, Galanis et al. (2023) [3] revealed that nearly two-thirds of nurses were considered quiet quitters. Moreover, these results were supported by Galanis et al. (2024) [35], who demonstrated that the mean value of the quiet quitting scale was neutral, which indicated that more than three-quarters of nurses were quiet quitters. This may be attributed to a lack of nurses’ managerial support, a lack of appreciation, and a lack of career development opportunities.

Furthermore, the average mean score of the participant nurses’ loyalty scale was low. The current study results were consistent with Ahmed et al. (2017) [36], who revealed that staff nurses had a higher intention to leave their hospital and the profession and had a moderate level of total organizational loyalty. This can be attributed to a stressful work environment and a lack of adequate support, which may cause nurses to feel overwhelmed and ultimately seek job opportunities elsewhere.

Additionally, the average mean score of the participants’ intention to leave scale was neutral. The current study results were also consistent with Ahmed et al. (2017) [36], who revealed that staff nurses had a higher intention to leave their hospital and the profession. Also, these results were supported by Galanis et al. (2023) [3], who revealed that more than two-fifths of nurses experienced high levels of turnover intention. This can be referred to as toxic leadership, managerial practices, a lack of a reward system, and poorly applied retention strategies and staff development opportunities.

There was a statistically significant direct effect of perceived quiet firing on nurse loyalty, which supported hypothesis I. These results agreed with the study conducted by Oquendo et al. (2024) [20], who demonstrated that quiet firing erodes organizational loyalty. This may be because quiet firing results in a lack of recognition for nurses’ contributions. This can lead to feelings of being ignored or neglected, causing frustration. When nurses feel unappreciated or invisible, they may be motivated to pursue other job opportunities where their efforts are acknowledged and valued.

Our study found that nurse loyalty and quiet quitting were negatively correlated, which supported hypothesis II. This result was supported by the study performed by Toska et al. (2025) [37], who revealed that quiet quitting was negatively correlated with employees’ loyalty. This result was supported by the study done by Karadas & Çevik (2024) [32], who found that the quiet quitting had a strong negative correlation with employee loyalty. This may be due to nurses who quietly quitted required often lack the motivation to contribute to the organization in a meaningful way, and this lack of initiative reduces their loyalty.

Moreover, the current study demonstrated that the quiet quitting scale and quiet firing scale were positively correlated. When healthcare professionals perceive that they are being quietly fired (e.g., marginalized, denied opportunities, or unsupported), they may respond with disengagement behaviours associated with quiet quitting. This contrasts with Karadas & Çevik (2024) [32], who argued that experiencing quiet firing may deter quiet quitting, possibly due to increased anxiety about job security or efforts to regain favour. The current study, however, implies that perceived organizational neglect may foster disengagement rather than reduce it.

Furthermore, the quiet quitting intention and perceived quiet firing scale were positively correlated. This result agreed with the study conducted by Oquendo et al. (2024) [20], who revealed that quiet quitting and quiet firing are interdependent behaviors characterized by a potential mutual causation relationship. This could be explained by managerial practices of silent firing that disregard employees, show them no appreciation, and foster a hostile workplace that causes nurses to quietly quit.

The current study revealed that the intention to leave scale and both the participants’ quiet quitting intentions and perceived quiet firing were positively correlated. This result agreed with Kim & Sohn (2024) [38], who suggested that as quiet quitting increases, turnover intention also rises significantly. This may be because quiet quitting involves a psychological and emotional disengagement from the organization, which weakens their connection to their work and makes them more likely to leave. Additionally, Anand et al. (2024) [39], revealed that there was a positive correlation between quiet firing and nurses’ turnover intention. This can be attributed to passive actions by the manager that cause nurses to feel undervalued, unsupported, or even alienated. These indirect actions lead to negative emotional and psychological effects, which in turn, increase the likelihood of nurses’ intention to leave.

Besides that, there was a low statistically significant negative correlation between nurses’ loyalty intention to leave scale, indicating that higher loyalty is associated with a reduced likelihood of leaving the organization. This result supports hypothesis III. This finding supports the notion that organizational commitment acts as a stabilizing factor, potentially buffering against turnover intentions even when other negative workplace dynamics, such as quiet quitting or quiet firing, are present. It highlights the importance of cultivating loyalty as a strategic priority in nurse retention efforts.

Regarding the mediation analysis, there was a statistically significant direct effect of perceived quiet firing on intention to leave. This finding is supported by Ebrahim & Ahmed (2022) [40], who found a highly statistically significant direct correlation between the quiet firing practices and the intention of staff nurses to quit their jobs. Moreover, this is consistent with the study performed by Katırcıoğlu (2024) [41], who reported that quiet firing creates arduous and disagreeable working conditions that impel employees to resign from their jobs. This may be attributed to nurses’ perception that they are useless and not participating in the decision-making process, and feeling they are unwanted in the organization.

Furthermore, there was a statistically significant direct effect of perceived quiet firing on nurses’ quiet quitting intentions. This result disagreed with the study conducted by Oquendo et al. (2024) [20], who revealed that quiet quitting and quiet firing are interdependent behaviors characterized by potential mutual causation rather than a clear cause-and-effect relationship. This may be because quiet firing can leave nurses feeling powerless regarding their professional future. When they sense that their efforts are being undermined or that their job performance is not genuinely recognized, they may withdraw and stop putting in extra effort. In this context, quiet quitting becomes a coping mechanism to manage the feelings of being devalued.

Moreover, the current study revealed that there was a statistically significant direct effect of quiet quitting intentions on intention to leave. These results were supported by the study done by Gün et al. (2025) [42], who demonstrated that turnover intention had a significant positive direct effect on the quiet quitting intention of nurses. This may stem from the fact that nurses who engage in quiet quitting are often dissatisfied with their roles and seeking change. If their concerns and needs remain unaddressed by the organization, they are more likely to leave for a workplace that provides better support, recognition, work-life balance, and opportunities for career growth.

Additionally, the current study revealed that there was a statistically significant direct effect of quiet quitting intentions on loyalty. This result was supported by the study performed by Toska et al. (2025) [37], who found that quiet quitting significantly undermines employees’ loyalty. This could be because nurses who are quietly fired are more likely to quietly depart, which lowers their dedication and loyalty and reflects on their willingness to stay with the organization.

Furthermore, there was a statistically significant indirect effect of perceived quiet firing on nurses’ intention to leave and loyalty when the perceived quiet firing acts as a mediator variable. This result supported hypothesis IV. This result was different from the study conducted by Bayer & Cankaya (2022) [43], who revealed that nurses’ perceptions of organizational loyalty had a direct negative effect on their turnover intention. Also, Ahmed et al. (2017) [36], revealed that there was a statistically significant direct negative correlation between organizational loyalty and intention to leave the nursing profession. This may be attributed to various factors and psychological processes that influence how loyalty to an organization affects a nurse’s likelihood of leaving. Also, quiet firing covert instance of organizational neglect that undermines the elements that make nurses feel a sense of loyalty to their jobs and increases their turnover intention.

## Conclusion

The results reveal a strong, significant positive correlation between quiet quitting intentions and perceived quiet firing. Nurse loyalty negatively correlates with quiet quitting, quiet firing, and the intention to leave. Notably, perceived quiet firing directly influences the intention to leave, moderated by nurse loyalty. This paper provides valuable insights into how quiet firing affects nurses’ likelihood of quiet quitting, their loyalty, and their intention to leave.

### Implications for nursing policy

When considering the association between quiet quitting, intention to leave, and nurses’ loyalty, it’s essential to offer recommendations that address the root causes of disengagement, burnout, and turnover and foster an environment that encourages engagement, job satisfaction, and retention.

Policy implications to increase nurses’ loyalty by increasing nursing participation in hospital committees, promotion opportunities, implementation of professional practice models, and use of mentorship programs to competitive compensation and career development opportunities. Implement clear and fair recognition programs that reward nurses for their hard work, dedication, and contributions. This could include formal recognition programs, performance bonuses, or non-financial rewards such as public acknowledgment.

Nurse managers should provide sufficient resources (e.g., staffing levels, training, access to equipment) and emotional support (e.g., mentorship, peer support, counseling services), implement flexible scheduling, provide adequate breaks, and encourage nurses to take time off to avoid burnout.

A multi-party review technique, such as a 360-degree personnel appraisal, should be used to avoid poor leadership and human resources practices. Shared governance strategies are used when minimizing expenses or downsizing is required, employees should be notified, and accountability and fairness should be given priority.

Nurses often face high stress levels due to the nature of their work. Implementing comprehensive well-being programs that include mental health support, stress management workshops, and initiatives focused on nurses’ physical health (e.g., wellness programs, ergonomic assessments) can promote long-term well-being, reduce burnout, and improve retention and loyalty.

Another human resources policy is needed to increase awareness through regular surveys, focus groups, and one-on-one meetings to understand nurses’ concerns, needs, and satisfaction levels. Actively listen to their feedback and make improvements based on their input.

### Limitations

Quiet quitting and intention to leave are processes that unfold over time, and understanding these behaviors in the context of nurses’ loyalty may not be fully captured through the current quantitative dynamics. Also, the scope of the study was limited to a single hospital. Also, Nurses’ perceptions of their job, organizational culture, and loyalty can differ widely based on their personal experiences, background, and individual coping mechanisms. One nurse’s definition of “quiet quitting” may differ from another’s. Future researches should aim for larger, more diverse samples, use mixed-methods approaches, and control for confounding variables.

## Data Availability

The datasets generated and/or analyzed during the current study are not publicly available due confidentiality but are available from the corresponding author on reasonable request.
